# Antihypertensive Peptides from Ultrafiltration and Fermentation of the Ricotta Cheese Exhausted Whey: Design and Characterization of a Functional Ricotta Cheese

**DOI:** 10.3390/foods10112573

**Published:** 2021-10-25

**Authors:** Erica Pontonio, Marco Montemurro, Gina Valeria De Gennaro, Valerio Miceli, Carlo Giuseppe Rizzello

**Affiliations:** 1 Department of Soil, Plant and Food Science, University of Bari Aldo Moro, via Giovanni Amendola 165/A, 70126 Bari, Italy; erica.pontonio@uniba.it (E.P.); marco.montemurro@uniba.it (M.M.); g.vale94@libero.it (G.V.D.G.); 2 ENEA Research Centre, Department for Sustainability, 72100 Brindisi, Italy; valerio.miceli@enea.it; 3 Department of Environmental Biology, Sapienza University of Rome, Piazzale Aldo Moro 5, 00185 Rome, Italy

**Keywords:** ricotta cheese exhausted whey, anti-ACE activity, fermentation, bioactive peptides, food by-products

## Abstract

Aiming at valorizing the ricotta cheese exhausted whey (RCEW), one of the most abundant by-products from the dairy industry, a biotechnological protocol to obtain bioactive peptides with angiotensin-I-converting enzyme (ACE)—inhibitory activity was set up. The approach was based on the combination of membrane filtration and fermentation. A *Lactobacillus helveticus* strain selected to be used as starter for the fermentation of the ultrafiltration protein-rich retentate (R-UF) obtained from RCEW. The fermented R-UF was characterized by a high anti-ACE activity. Peptides responsible for the bioactivity were purified and identified through nano-LC–ESI–MS/MS. The sequences identified in the purified active fractions of the fermented R-UF showed partial or complete overlapping with previously reported κ-casein antihypertensive fragments. The fermented R-UF was spray-dried and used to enrich ricotta cheese at different fortification level (1 and 5% *w*/*w*). An integrated approach including the assessment of the microbiological, chemical, functional, textural, and sensory properties was used to characterize the fortified products. A significantly higher anti-ACE activity was found in the ricotta cheese fortified with fermented R-UF as compared to the control and to the samples obtained with the unfermented R-UF fraction at the same levels of fortification. In particular, a 100 g portion of the ricotta cheese produced at 5% fortification level contained circa 30 mg of bioactive peptides. The fortification led to a moderate acidification, increased hardness and chewiness, and decreased the milk odor and taste of the ricotta cheese as compared to the control, while flavor persistence and sapidity improved.

## 1. Introduction

The management of food waste and by-products, due to the global increase of population and food consumption, is a challenge for the agri-food industry that faces growing economic costs for the treatment and/or disposal of waste and stringent environmental regulations [1].

Among different production activities [1], the dairy industry annually produces millions of tons of by-products, mainly represented by cheese whey (CW), which corresponds to the liquid fraction remaining after milk coagulation. Around 9–10 L of whey results from the production of 1 kg of cheese. Due to its consistent organic load [2,3], if discarded without treatment, CW creates significant problem for the environment. Besides disposal, CW is used for feed, and to a small extent as substrate for the recovery or the synthesis (through physical, chemical, and biotechnological processes) of molecules having nutritional and pharmaceutical potential. However, in many countries such as Portugal, Spain, Italy, and Turkey, CW is employed to produce whey-derived cheeses (e.g., requeijão, requesón, ricotta, and lor, respectively) [4,5,6]. Acidification and heating at 85–90 °C for 20–30 min of the CW, to allow coagulation and subsequent precipitation of whey proteins and separation of whey cheese mass, are used in the production of ricotta [7,8,9].

Although depending on the origin of the whey and the process employed, the whey cheese yield is lower than 4%, unless whey is previously concentrated. The liquid remaining after whey cheese separation (“cheese exhausted whey”, CEW) represents more than 90% of the original whey and it is the main by-product of the whey cheese production chain. CEW obtained from the ricotta production is called “Scotta” in Italy, and commonly defined as ricotta cheese exhausted whey (RCEW).

As is the case of CW, since it is characterized by high values of BOD (biochemical oxygen demand) and COD (chemical oxygen demand) (circa 50 and 80 g/L, respectively), CEW is considered highly polluting [9,10]. Indeed, if discarded into water sources, it reduces the dissolved oxygen, and represents a risk to aquatic life, as well as to the environment and human health [11]. Due to the high content of lactose (35–50 g/L), which cannot be digested by most animals without suffering from digestive disorders [9,12], the valorization of CEW represents a considerable problem. Moreover, small- and medium-size dairy industries often lack dimension to make the necessary investments for CEW valorization [2,8]. Nevertheless, CEW is considered as a source of functional and bioactive compounds, especially proteins and peptides [13,14,15].

The concentration processes required by the relatively low content of proteins in CEW and needed to ensure high hydrolysis yields, are some of the main problems impairing its industrial reuse [16]. Several methods, such as ultrafiltration, diafiltration, nanofiltration, ion exchange chromatography, electrophoresis, crystallization, and precipitation have been proposed to concentrate and separate proteins from other components contained in CW and CEW. These techniques could be applied for the recovery of the major compounds (β-lactoglobulin and α-lactalbumin), but also for minor compounds, such as lactoperoxidase, lactoferrin, immunoglobulins, as well as protein derivatives such as the biologically active peptides obtained by fermentation with either selected proteolytic microorganisms or the enzymatic or chemical hydrolysis of proteins [17].

In this work, a biotechnological protocol for obtaining bioactive peptides with ACE-inhibitory activity was set up by using a protein-rich fraction obtained from RCEW through a membrane ultrafiltration (UF) process [18] as substrate. A proper lactic acid bacteria (LAB) strain was selected to be used as a starter for the fermentation of RCEW. Bioactive peptides were purified and identified. The fermented RCEW active fraction was spray-dried and used to enrich ricotta cheese at different fortification level (1 and 5% *w*/*w*). Functional ricotta cheese, produced at a pilot plant level, was characterized by an integrated approach including the assessment of the microbiological, chemical, functional, textural, and sensory properties.

## 2. Materials and Methods

### 2.1. Microrganisms

The 5 strains of lactic acid bacteria (LAB) used in this study are listed in Table S1. Strains were previously isolated from cheeses: “Flor di capra” (sheep milk cheese) [19], Pecorino Umbro (sheep milk cheese) [19], Caciocavallo Pugliese [20], Parmigiano Reggiano [21], and fermented milk [22] and identified by partial sequencing of the 16S rRNA, recA, pheS, and rpoA genes. *Lacticaseibacillus casei*, *Lactobacillus delbrueckii* subsp. *bulgaricus*, *Lactobacillus helveticus*, and *Lactococcus lactis* strains were cultured on De Man, Rogosa, and Sharpe (MRS, Oxoid, Basingstoke, Hampshire, UK) at 37 °C for 24 h. *Streptococcus thermophilus* strain was grown on M17 containing 0.5% (*w*/*v*) lactose (Oxoid) at 37 °C for 24 h. All strains belong to the culture collection of the Department of Soil, Plant and Food Sciences (DiSSPA) of the University of Bari (Italy) and were maintained as stocks in 15% (*v*/*v*) glycerol at −80 °C.

### 2.2. RCEW Fermentation

Caseificio dei Colli Pugliesi (Santeramo, Italy) kindly provided the RCEW that was used as substrate for the starter selection. The RCEW (aliquot of 200 L) was pasteurized (72 °C for 15 s) immediately after ricotta cheese production using an industrial plate pasteurizer (Alfa Laval, Lund, Sweden). After pasteurization, the RCEW was cooled down at 4 °C in less than 16 min. No protein flocculation occurred during thermal treatment. The RCEW was then stored at 4 °C and fermented within 24 h from pasteurization.

Strains were singly used to ferment the RCEW. Cells were harvested by centrifugation (10,000× *g*, 10 min, 4 °C), washed twice in 50 mM phosphate buffer, pH 7.0, and re-suspended in RCEW at a final cell density of circa 7.0 log10 cfu/mL of sample. All the samples were incubated for 24 h at 30 or 37 °C, according to the strain used.

Values of pH prior and after the fermentation were measured using a pH meter M.507 (Crimson, Milan, Italy). The cell density of presumptive LAB was enumerated using either the MRS or M17 medium according to the strain inoculated (Table S1).

Plates were incubated under anaerobiosis (AnaeroGen and AnaeroJar, Oxoid) at 30 or 37 °C, according to the strain used, for 48 h. The concentration of peptides in RCEW and fermented RCEW was determined through the o-phtaldialdehyde (OPA) method as described by Church et al. [23]. Aiming at removing proteins and free amino acids, treatments with trifluoroacetic acid (TFA, 0.05% *w*/*v*) and dialysis (cutoff 500 Da) were performed, respectively. The total free amino acids (TFAA) concentration was determined using a Biochrom 30 series amino acid analyzer (Biochrom Ltd., Cambridge Science Park, UK) with a Li-cation-exchange column (0.46 cm internal diameter), by post column derivatization with ninhydrin, as described by Rizzello et al. [24]. Fermentations and analysis were carried out in triplicate.

### 2.3. RCEW ACE-Inhibitory Activity

ACE-inhibitory activity in the RCEW was analyzed prior and after the fermentation by the method reported first by Cushman and Cheung [25] and Wu and Ding [26] and modified by Rizzello et al. [27]. Compared to the original method, the new analytical procedure does not include the ethyl acetate extraction. Previously, it was successfully used for the analysis of protein hydrolysates [28]. Two hundred microliters of 5 mM hippuryl-L-histidyl-L-leucine (HHL) solution (obtained in a Na-borate buffer, pH 8.3, supplemented with 300 mM NaCl) were mixed with 60 µL of the peptides mixture and 40 µL of 100 mU/mL ACE (in a phosphate buffer at 10 mM, pH 7.0, supplemented with 500 mM NaCl). Water was used in the negative control instead of samples. Incubation of the reaction mixture was carried out at 37 °C for 60 min; to stop the reaction, 250 µL of 1 M HCl were added at the end of the incubation. The mixture was then analyzed by HPLC, using a Resource RPC C18 column (6.4 × 100 mm, particle size 15 µm; GE Healthcare Bio-Sciences AB), aiming at quantifying separately the product and hippuric acid (HA) from HHL. In detail, elution was carried out by using a mobile phase composed of water and acetonitrile (CH_3_CN) containing 0.05% TFA. The flow rate was 1 mL/min, while the detection was carried out at 228 nm. The analysis was carried out by setting a linear CH_3_CN gradient from 5 to 46% (between 16 and 62 min). The inhibition activity was calculated using the following equation:

inhibition activity (%) = ((Pc − Ps)/(Pc − Pb))100,(1)

where Pc is the HA-peak area of the control, Ps is the HA-peak area of the reaction mixture (sample), and Pb is the HA-peak area of the reaction mixture without ACE.

### 2.4. RCEW Fractionation and Characterization

RCEW was fractionated at the pilot plant of the ENEA (Brindisi, Italy), delivered under refrigerated conditions (4 ± 2 °C), and processed within 24 h from production [29].

A multistep fractionation process based on a separative membrane process previously proposed by Raho et al. [18] was applied to RCEW, aiming at collecting separately a protein-rich fraction, a lactose-rich fraction, and ultrapure water. From each fractionation step, two fractions were obtained: (i) aqueous permeate (P) containing the molecules able to cross the membrane and (ii) retentate (R) containing a residual part of these and all the molecules unable to cross the membrane. The retentate of the first fractionation step (R-UF), rich in proteins, was collected and used in this work. The ultra-filtration (UF) was carried out by a prototypal system equipped with a spiral wound PESH (polyethersulfone) membrane (30 kDa cutoff) ISUH030 4040 C1 (Microdyn-Nadir, Wiesbaden, Germany), with a spacer of 44 mil (1.117 mm) and an area of 6 m^2^. The process was carried out at the flow rate of 3000 L/h, at 14 °C. The transmembrane pressure (TMP) corresponded to 3.47 bar while permeate flow was 15.4 L/h·m^2^.

Fraction recover was expressed as % *v*/*v*, while the volume concentration ratio (VCR) was calculated as the ratio between initial feed volume and retentate volume. Before analysis, all the RCEW fractions obtained were stored at −20 °C. All the analyses were performed in triplicate.

The pH of the RCEW and R-UF was determined as reported above. A standard ISO 8968-1 Kjeldahl-based method [30] was used to quantify the total nitrogen (TN). The calculation of total proteins was carried out by using 6.38 as the conversion factor. Sugars (glucose, galactose, and lactose) were determined by HPLC analysis, using an ÄKTA purifier HPLC (GE Healthcare) as reported by Verni et al. [31]. Commercial standards of lactose, glucose, and galactose (Sigma Aldrich, Milano, Italy) were used for the identification of the sugars and for obtaining the calibration curves. The TFAA concentration was determined using a Biochrom 30 series amino acid analyzer as described above. The international standards methods Gerber [32] and AOAC 945.46 [33] were used to quantify fat and ash, respectively. The sodium chloride content in the R-UF was measured by a Sherwood 926 chloride-analyzer (Sherwood Scientific, Cambridge, UK).

### 2.5. R-UF Fermentation

The R-UF was fermented with *L. helveticus* PR4, selected for its ability to increase ACE-inhibitory activity of the RCEW in the preliminary screening. In details, the R-UF was inoculated as described above (final cell density of circa 7.0 log10 cfu/mL) and fermentation was carried out at 37 °C for 24 h. Lactose, protein and peptide concentrations, and ACE-inhibitory activity were determined as described above.

### 2.6. Spray-Drying

Unfermented and fermented R-UF were spray-dried using a pilot plant spray dryer (Mini Spray Dryer B-290, Büchi, Switzerland) at a drying rate of 1.0 L/h. The spray-drying system included a fluid nozzle (0.7 mm diameter) to atomize liquid feed into fine droplets, and a drying chamber (16.5 cm diameter, 45 cm height) in which atomized liquid was dried by a flow of hot air. Two cyclone separators were used for collecting powder. The first separator collects coarser particles, and the fine and ultrafine particles were recovered by the first and the second separators, respectively. A peristaltic pump was used to feed the system with a controlled flow rate. The inlet and outlet temperatures of the spray dryer system were 200 and 180 °C, respectively. For each spray-drying experiment, 50 to 100 mL of sample (preconditioned at 25 °C) was pumped with a feed flow rate fixed at 10 mL/min. Pressure ranged from 5 to 8 bar. Dried powders were collected in the glass bottle connected to the separators and then stored until further analysis in airtight containers.

### 2.7. Purification and Identification of the Bioactive Peptides

An aliquot of fermented R-UF corresponding to 15 mg of peptides was automatically fractionated (2 mL per fraction, 33 fractions for each run) by reversed-phase fast performance liquid chromatography (RP-FPLC), using a Resource RPC column and ÄKTA FPLC equipment, with the UV detector operating at 214 nm (GE Healthcare Bio-Sciences AB, Uppsala, Sweden). Elution was carried out using a mobile phase composed of water and acetonitrile (CH_3_CN), containing 0.05% TFA (1 mL/min flow rate). The analysis was carried out using a gradient elution (CH_3_CN concentration in mobile phase was increased from 5 to 46% between 16 and 62 min, and from 46 to 100% between 62 and 72 min).

Fractions were freeze-dried to remove solvents and re-dissolved in sterile water to determine the peptide concentration through the OPA method. Each fraction was also subjected to the ACE-inhibitory assay as reported above.

The peptides contained in the fractions with the highest ACE-inhibitory activity were further purified and identified. The analysis was performed by nano-LC–ESI–MS/MS (nano-liquid chromatography–electrospray ionization–mass spectra/mass spectra), by using a ion trap mass spectrometer (Finnigan LCQ Deca XP Max, Life Technologies GmbH, Darmstadt, Germany, ThermoElectron). A nano-ESI interface was used. MS spectra were automatically recorded through Xcalibur software (Life Technologies GmbH, Darmstadt, Germany, ThermoElectron), in positive ion mode, following the manufacturer’s instrument settings. The software program BioWorks 3.2 (Life Technologies GmbH, Darmstadt, Germany, ThermoElectron) was used for MS/MS spectra processing. Peptides were identified through the Mascot search engine (Matrix Science, London, UK) using the NCBIProt database (National Centre for Biotechnology Information, Bethesda, MD, USA). Settings used for peptides identification were: instrument type, “ESI-trap”; enzyme, “none”; peptide mass tolerance, ±0.1%; fragment mass tolerance, ±0.5 Da. Results were screened as described by Chen et al. [34]. Validated peptide sequences explained all the major peaks in the MS/MS spectrum.

### 2.8. Ricotta Production

Experimental ricotta cheese was produced at industrial level at “Caseificio dei Colli Pugliesi” (Santeramo, Bari, Italy) from December 2019 to March 2020. The whey, collected from mozzarella making, was strained and then heated at 85–90 °C under stirring conditions. When proteins started to flocculate, stirring was stopped to promote the formation of aggregates. The curd was then scooped, moved into perforate molds, and kept for 30 min in a cool room (4 °C) for draining. Then, 5 kg portions of the clot were gently mixed with the spray-dried R-UF and fermented R-UF (fR-UF). Five different types of ricotta cheese were obtained: ricotta cheeses supplemented with 1% and 5% (*w*/*w*) of the spray-dried fermented fR-UF (RCf1 and RCf5); ricotta cheeses supplemented with 1% and 5% (*w*/*w*) of the spray dried unfermented R-UF (RC1 and RC5), and a control sample produced without a R-UF addition. After R-UF addition, ricotta cheeses were divided into 100 g portions, placed in perforate molds, and stored at 4 °C until analysis. Three ricotta productions were done on three different days.

### 2.9. Ricotta Characterization

The pH values of ricotta samples were determined as reported above. Ricotta samples were homogenized in a home pulverizing machine. The ash contents of whey samples were determined according to AOAC [33]. The determination of fat (% *w*/*w*) was carried out using the Gerber–Van Gulik method [32]. The solid non-fat content in the whey was determined by using the following formula: total solid% − fat%. The total nitrogen was determined using the Kjeldahl method and converted into protein percentage using the conversion factor 6.38 (the nitrogen-to-protein conversion factor for milk and dairy products) [30]. The moisture content and total dry matter of ricotta were determined by drying samples on a BRASIMET Model ESE 35 stove at 105 °C for 12 h to achieve a constant weight [35].

For the microbial analysis, 10 g of each ricotta cheese were suspended in 90 mL of sterile sodium chloride (0.9% *w*/*v*) solution and homogenized in a BagMixer 400P (Interscience, St Nom, France) at room temperature to enumerate the microbial cell number. Serial 10-fold dilutions were then plated into Plate Count Agar (PCA, Oxoid, Basingstoke, Hampshire, UK) supplemented with cycloheximide (0.1 g/L), MRS (Oxoid) supplemented with cycloheximide (0.1 g/L), Violet Red Bile Glucose Agar (VRBGA, Oxoid), and Slanetz and Bartley Agar (Oxoid) used to enumerate total thermophilic bacteria, presumptive thermophilic LAB, *Enterobacteriaceae* and enterococci, respectively. Except for *Enterobacteriaceae*, which were enumerated after 24 and 48 h, all microbial groups were counted after 48 h incubation at 37 °C. Yeasts and molds were enumerated on malt extract (Oxoid) and wort agar (Oxoid), respectively, after incubation for 48 h at 25 °C.

Water-soluble extracts of the ricotta samples were prepared according to the method of Kuchroo and Fox [36] with some modifications. Ricotta samples were mixed with a phosphate buffer (0.05 M and pH 7) in a ratio 1:2 (*w*/*v*) and homogenized in a BagMixer 400P (Interscience, St Nom, France) at room temperature using a stomacher (PBI International, Milano, Italia). Then, samples were incubated at 40 °C for 1 h under stirring condition (75 rpm) and centrifuged at 4500 rpm for 30 min at 4 °C. The supernatant was filtered (0.22 µm), the pH adjusted with 0.1 M HCl to pH 4.6, and centrifuged at 11,200× *g* for 10 min at 4 °C.

The 70% ethanol-soluble extract was prepared from the water-soluble extract by adding absolute ethanol to a final ethanol concentration of 700 mL/L. The mixture was held for 30 min at 20 °C and then centrifuged (3000× *g* for 30 min). The supernatant was filtered through Whatman no. 1 filter paper. Rotary evaporation under vacuum (model no. RE100, Bibby Sterlin Ltd., Stone, UK) at 30 °C was used for removing ethanol from the extract. The ethanol-soluble and -insoluble extracts were resuspended in water, lyophilized, and stored at 4 °C until further analyses.

The peptide profile of the ethanol-soluble fraction was determined by RP-HPLC using a Waters 626 system equipped with a Waters 600 controller and a Waters 717 plus autosampler (Waters Corp., Milford, MA, USA). Guard and analytical columns used were Nucleosil C8 (5 μm particle, 300 Å pore size), with 4.6 × 10 mm × mm and 4.6 × 250 mm diameter, respectively (Macherey-Nagel GmbH, Duren, Germany). A Varian 9050 UV–Vis Detector (Varian Inc., Walnut Creek, CA, USA) was used for detection at 214 nm. Chromatograms were recorded by the Millennium software program (Waters). The chromatographic conditions were as follows: solvent A, 1 mL/L TFA (Sigma, St. Louis, MO, USA) in deionized, HPLC-grade water (Milli-Q system, Waters Corp.); solvent B, 1 mL/L TFA in CH_3_CN (HPLC-grade, Labscan Ltd., Dublin, Ireland). Samples (4 mg/mL) were dissolved in solvent A and filtered through 0.45 μm cellulose acetate filters (Sartorius GmbH, Gottingen, Germany). Aliquots of 40 μL were loaded onto the column and eluted (0.75 mL/min flow rate) using the gradient previously proposed by Shakeel-Ur-Rheman et al. [37]

An ACE-inhibitory assay was performed on the ethanol-soluble fraction as described above.

### 2.10. Sensory Analysis

Sensory analysis of ricotta samples was carried out by 10 semi-trained panelists, with an equal distribution of men and women ranging in age between 25 and 40 years. Before the analysis, 30 panelists underwent a minimum of 5 h of training and were chosen based on their capacity to distinguish and describe the sensory attributes listed in Table S2.

The 5 h training was performed as follows: (i) the assessors attended a short class on the type of ricotta cheese to be evaluated; (ii) a clear definition of sensory attributes was provided along with the presentation and evaluation of the different physical references (Table S2). Physical and written definitions were available to panelists during all sensory evaluations.

The lexicon consisted of nineteen attributes and followed the Lawless and Heymann [38] guidelines. Ricotta samples were randomly coded and served at 18 to 20 °C in portions of 20 g, together with non-salted table biscuits and still water, to panelists placed separately in rooms for unbiased evaluation of sensory attributes. Ricotta samples were scored from 1 (very unpleasant) to 7 (excellent and fresh) where the value 4 was set as the minimum threshold of sensory acceptability, in agreement with what is reported in the literature for the sensory analysis of dairy products [39,40]. The study protocol followed the ethical guidelines of the sensory laboratory. A written informed consent was obtained from each participant.

### 2.11. Texture Profile Analysis of Ricotta

The textural properties of ricotta were evaluated at room temperature (18 ± 2 °C) by using a FRTS-100N texture analyzer (Imada, Toyohashi, Japan) equipped with a cylinder probe FR-HA-30J on ricotta cheese specimens of 60 ± 1 g. The instrument settings were the following: test speed of 1 mm/s, 30% deformation of the sample, and two compression cycles while the parameters evaluated were hardness (expressed as the maximum force at first compression), cohesiveness (ratio of the areas of the second and the first compression peak), springiness (height of the product on the second compression divided by the height of the first peak), and chewiness (hardness × cohesiveness × springiness).

### 2.12. Statistical Analysis

All the chemical, microbiological, and physical analyses were carried out in triplicate for each batch of sample. Data were subjected to one-way ANOVA; pair-comparison of treatment means was achieved by Tukey’s procedure at *p* < 0.05, using the statistical software package Statistica 12.5 (StatSoft Inc., Tulsa, OK, USA).

## 3. Results

### 3.1. LAB Selection

The five strains of LAB were singly inoculated into the RCEW. The acidification and proteolytic activity (quantified as concentration of peptides released during fermentation) were used as selection criteria together with the capability to enhance the ACE-inhibitory activity of the fermented RCEW. The capability of growth of the strain was also assessed through the enumeration of the presumptive LAB. Overall, the pH of fermented RCEW was from 0.5 to 1 unit lower than the CT (Table 1).

Growth from 0.5 (*L. lactis* DIBCA2) to 2 (*L. helveticus* PR4) log10 cfu/mL were observed during the incubation of the RCEW. A significant (*p* < 0.05) and markedly lower cell density was found in CT (3.9 ± 0.1 log10 cfu/mL). Increases from circa 40 (*L. lactis* DIBCA2) to 125% (*L. helveticus* PR4 and *S. thermophilus* CR12) were found in fermented RCEW (Table 1). Nevertheless, the highest peptide concentration did not correspond to the highest ACE-inhibitory activity. Indeed, the RCEW fermented with *L. lactis* DIBCA2 and *S. thermophilus* CR12 did not show (*p* > 0.05) significant increases of the ACE-inhibitory activity compared to the CT (Table 1). Significantly (*p* < 0.05) higher values (2 and 4 times) were found for *L. casei* FC13 and *L. delbrueckii* subsp. *bulgaricus* B15Z, while an activity circa 10 times higher than CT was found for the RCEW fermented with *L. helveticus* PR4. According to the data reported above, *L. helveticus* PR4 was selected and used for further experiments.

### 3.2. RCEW Fractionation

The RCEW was subjected to ultrafiltration, as previous described by Raho et al. [18]. The RCEW’s initial pH was 5.2 (Table 2).

Most of the nitrogen and fat of the RCEW was retained in the protein-rich retentate (R-UF), as a result of the separation process. R-UF corresponded to the 20.3% *v*/*v* of the processed RCEW, with a volume concentration ratio (VCR) of 4.92.

The protein concentration of the fraction, estimated based on the TN content (290 ± 4 mg/L) corresponded to 1.85 ± 0.05% *w*/*v*, while fat was 0.75 ± 0.04% *w*/*v*. The retentate was moreover characterized by a relevant lactose content (5.19 ± 0.22% *w*/*v*) (Table 2).

As expected, a low TN concentration characterized the UF permeate (P-UF). The remaining TN corresponded to organic compounds having molecular mass lower than 30 kDa (mainly peptides and free amino acids, FAA). In particular, the TFAA concentration in P-UF corresponded to 516 mg/L. The ash concentration of the permeate was significantly (*p* < 0.05) higher than that of the RCEW since fractionation and separation of the R-UF allowed a partial water removal.

### 3.3. Fermentation and Characterization of the Protein-Rich RCEW Fraction

Aiming at releasing the highest concentration of bioactive peptides, *L. helveticus* PR4 was used to ferment the R-UF fraction obtained from the RCEW. The main characteristics of the R-UF (prior the fermentation) and fR-UF (after the fermentation) are summarized in Table 3.

A total bacteria cell density of 2.65 log10 cfu/mL characterized the R-UF before the inoculum. During fermentation, *L. helveticus* PR4 reached a cell density of 8.8 ± 0.4 log cfu/mL, while the pH decreased by circa 1.5 units (Table 3). The fR-UF was characterized by concentrations of peptides and TFAA 50 and 18 times higher than the R-UF. The concentration of the lactose was also significantly (*p* < 0.05) lower (circa 33%). The fR-UF was characterized by an ACE-inhibitory activity of 88.2 ± 1.1% (Table 3).

The spray-drying process allowed to recover circa 6.95 ± 0.10 and 7.80 ± 0.08% (*w*/*v*) powder from the R-UF and fR-UF, respectively, both having circa 7% moisture. Spray-dried R-UF and fR-UF were characterized by similar TN (3.54 ± 0.12 and 3.56 ± 0.09 g/kg, respectively). According to the conventional calculation (applying the conversion factor 6.38 to TN), the protein concentration theoretically corresponded to 22.58% and 22.71% (*w*/*w*) of the spray-dried R-UF and fR-UF, respectively. Nevertheless, this estimation is approximate, and the analysis revealed peptides and TFAA concentrations markedly higher in the fR-UF (46.44 vs. 0.83 g/kg of peptides and 12.80 vs. 0.62 g/kg of TFAA), due to the LAB proteolysis occurring during fermentation. As expected, the spray dried R-UF and fR-UF were characterized by relevant concentration of lactose (65.5 and 50.04%, respectively) and fat (circa 9.0% for both). The LAB density in the R-UF and fR-UF was <10^4^ cfu/g.

### 3.4. ACE-Inhibitory Peptides

**Figure 1:**
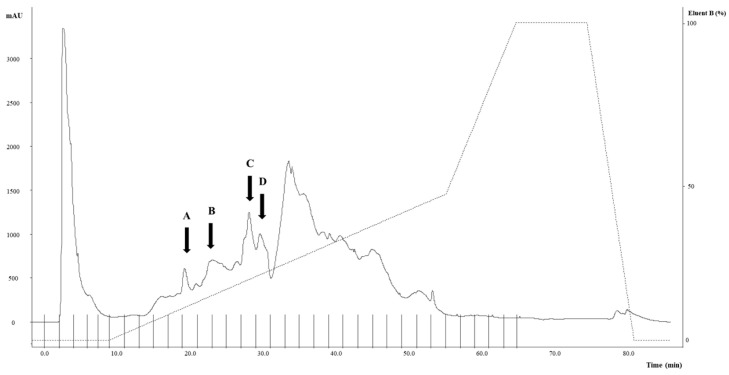
RP-FPLC chromatogram (UV detector 214 nm) showing the WSE fractions obtained from the purification of the fermented R-UF. The gradient of eluent B is represented by the dashed line. A–D refer to the fractions with anti-ACE activity.

The fR-UF was fractionated by RP-FPLC; then, fractions were freeze-dried, resuspended in 1 mL of sterilized water, and assayed for assessing the ACE-inhibitory activity. Four of the thirty fractions (Figure 1) showed ACE-inhibitory activity, corresponding to 68.11 (fraction A), 54.49 (fraction B), 35.05 (fraction C), and 36.55% (fraction D) (Figure 1).

The peptides concentration of the fractions was 0.32, 0.43, 0.52, and 0.92 mg/mL for A, B, C, and D, respectively. Two out of four extract fractions contained a mixture of peptides. Indeed, fractions A and D contained four and two peptides, respectively. The identified peptides, having 12–25 amino acid residues, were characterized by molecular mass ranging from 1304.4 (AQPTDASAQFIR) to 2657.9 Da (NQDKTEIPTINTIASGEPTSTPTIE) and hydrophobic ratio between 32 (DETHLEAQPTDASAQ) and 49% (VIESPPEINTVQVTSTAV and AQPTDASAQFIR). The net charges at pH 7 (antimicrobial peptide calculator and Predictor-APD3) [41] was negative for all peptides except for RHPYFYAPELLYYANK and AQPTDASAQFIR having positive and neutral values, respectively (Table 4).

### 3.5. Ricotta Cheeses Characterization

Microbiological properties of the ricotta cheeses were also investigated. Overall, no significant (*p* > 0.05) differences were found among samples. The microbial cell density ranged from circa 2.5 ± 0.3 (yeast, molds, and *Enterococcus*) to 3.5 ± 0.2 (LAB and *Enterobacteriaceae*) log10 ufc/g. Total bacteria cell density was 3.8 ± 0.3 log10 ufc/g.

Biochemical and nutritional properties of the ricotta cheeses are summarized in Table 5.

Overall, the fortification affected some of the characteristics of the samples with the RCf showing the highest differences. As expected, the pH and TTA values were significantly (*p* < 0.05) lower in RCf1 and RCf5 compared to the RC samples, with the lowest value found in RCf5 (Table 5). The highest level of fortification (5% *w*/*w*), regardless the fermentation, led to lower moisture (circa 4%) and carbohydrates concentration significantly (*p* < 0.05) higher than the corresponding samples fortified with 1% (*w*/*w*) of either the R-UF or fR-UF (Table 5). In addition, the protein content was slightly affected by the fortification, with RCf5 showing the highest concentration (circa 12% higher than RC).

A weak ACE-inhibitory activity was found in RC, RC1, and RC5 (from 7.2 to 7.5%), while significant (*p* > 0.05) higher values were found in RCf1 and RCf5 (Table 5). The highest anti-ACE activity (circa nine times higher than RC), was found in RCf5.

Textural profile analysis showed slight differences among samples. The fortification with 1% R-UF or fR-UF did not cause significant (*p* > 0.05) differences in the hardness, compared to control RC. Nevertheless, when 5% (*w*/*w*) R-UF was added to the formulation (RC5), a marked increase in the hardness, cohesiveness, and chewiness was found (Table 5). RCf5 had a similar (*p* < 0.05) hardness compared to that of RC and a lower hardness and chewiness compared to those of the corresponding RC5 (Table 5).

### 3.6. Sensory and Textural Characteristics of Ricotta Cheese

The sensory data of the ricotta cheeses are summarized in Table 6.

According to the visual aspect, the fortification led to higher values of both color intensity and homogeneity, especially when the fR-UF was used. Moreover, the fortification led to a decrease of the milk odor, while increasing the acidic flavor. Similar data were found when the flavor was analyzed. Bitterness and off-flavors were almost not found in any of the sample analyzed. However, the persistence of flavor and sapidity was higher in fortified ricotta cheeses than in the RC, with the highest value found in RCf5. When tested, the main difference found between fortified and control ricotta cheeses was in terms of wetness, which was significantly lower than that of the control. The difference was higher when the fR-UF was used (Table 6).

## 4. Discussion

The dairy industry produces RCEW as one of its most abundant by-products, which is an inexpensive substrate rich in nutrients such as proteins (mainly in a denatured state), soluble peptides, oligosaccharides, lactose, non-protein nitrogen, hydro-soluble vitamins, several minerals, and free amino acids [18,42,43]. Although RCEW has a similar composition to CW, the concentration of its constituents is markedly lower, except for the ash content, often affected by acid and salt added to improve the whey proteins flocculation and aggregation and the ricotta yield [43]. Due to the same reasons (e.g., the addition of sodium bicarbonate to promote flocculation), the RCEW pH is higher than that of whey. Fat is almost completely removed with ricotta production [43]. Here, the multi-step fractionation allowed to retain the fat in the UF retentate and the separation of two fractions: one rich in protein (1.85%, *w*/*v*), which can be easily subjected to the recovery of whey proteins to be used as food and feed supplements [18], and one rich in lactose.

RCEW proteins have a globular structure, with a uniform distribution of non-polar, polar, and charged groups; they include β-lactoglobulin, α-lactalbumin, immunoglobulins, serum albumin, lactoferrin, lactoperoxidase, and several enzymes. Other protein components such as glycomacropeptide or caseinomacropeptide, which are released from κ-casein in the first step of enzymatic curdling are also present [1].

Food proteins usually contain, encrypted into their sequences, several biologically active peptides, inactive until bound into the primary structure [44]. Once released through enzymatic proteolysis and/or microbial fermentation, the free forms of the peptides demonstrate health effects in the gut or after systemic absorption into blood circulation.

Bioactive peptides have been reported to lower blood sugar, serum cholesterol, blood pressure, inhibit microbial growth, and hinder cancer development [45]. Among the high number of active sequences identified and characterized from plant and animal sources, antihypertensive peptides deriving from caseins and whey proteins were deeply investigated [46,47]. It was indeed reported that existing synthetic antihypertensive drugs have several side effects, thus pushing the scientific community towards the research of antihypertensive peptides as alternative therapeutics to control systemic blood pressure and to prevent cardiovascular diseases [48]. The key enzyme involved in blood pressure regulation is angiotensin-I-converting enzyme (ACE), a transmembrane metallopeptidase presents in many tissues (lung, thoracic aorta, heart, kidney, and liver) and biological fluids [49]. ACE hydrolyses the decapeptide angiotensin I into the octapeptide angiotensin II, which binds to AT1 receptors on vascular smooth muscles and endothelial cells leading to vasoconstriction and an increase of the blood pressure. Angiotensin II also inactivates the endothelium-dependent vasodilator bradykinin [50]. The inhibition of the ACE activity is therefore the target for antihypertensive agents. Whey proteins such as β-lactoglobulin, α-lactalbumin, bovine serum albumin, and immunoglobulin exhibit diverse physiological functions and their hydrolysates have been shown to have ACE inhibitory activity [46,51]. It was moreover demonstrated that RCEW is a good source of anti-ACE peptides, that can be successfully released by different proteolytic enzymes [52].

Many LAB have been shown to degrade milk proteins to release bioactive peptides during the fermentation occurring in dairy processes. Indeed, LAB are characterized by an efficient proteolytic system able to hydrolyze proteins of the growth matrix into small peptides (2 to 40 amino acid chains) and free amino acids [53,54]. Thanks to their proteolytic activity, LAB strains of the species *Lactobacillus acidophilus*, *Levilactobacillus brevis*, *Ligilactobacillus animalis*, *Lactococcus lactis, Lactobacillus helveticus,* and *Lactiplantibacillus plantarum* were successfully used as microbial catalysts for producing ACE-inhibitory peptides [22,55,56,57].

In this work, LAB strains previously isolated from dairy products and already selected for their capability to release ACE-inhibitory molecules [19,20,21,22,57] were used as starters for the fermentation of the RCEW. Among the five strains used, *L. helveticus* PR4 was selected since it was able to improve the ACE-inhibitory activity of the fermented RCEW, although *S. thermophilus* CR12 led to the release of a higher total amount of peptides.

Due to its high proteolytic activity, lactic acid production, rate of milk acidification, and ability to improve the flavor and texture, strains of *L. helveticus* are often used to produce fermented milk beverages and hard cheeses [58]. The *L. helveticus* strains exhibit strong extracellular proteinase activity and capacity to release relevant amount and types of different peptides during milk fermentation [57,59]. The number of cell envelope proteinases (CEP) varies among *L. helveticus* strains ranging from one to four enzymes [60], such as the pattern of the cytoplasmic peptidases [47]. Several *L. helveticus* strains were previously selected for their capability to release anti-ACE peptides from caseins [58].

In this research work, a *L. helveticus* PR4 strain was employed as selected starter for the fermentation of the R-UF fraction derived from RCEW through membrane separation, confirming the potential of the substrate to be enriched in antihypertensive peptides. Aiming at identifying the peptides responsible for the bioactivity, the fermented R-UF was fractionated by FPLC and the active subfractions characterized by the highest anti-ACE activity, subjected to a purification and nano-LC–ESI–MS/MS analysis.

Eight peptides were identified in the four active fractions, all characterized by a molecular mass ranging from 1.4 to 2.6 kDa. Two of the purified fractions contained mixtures of different peptides. Three of the identified peptides derived from the hydrolysis of the κ-casein. TIASGEPTSTPTTEA and NQDKTEIPTINTIASGEPTSTPTIE, which were identified in partially purified fractions A (eluted in 12% eluent B) and D (eluted in 22% eluent B), respectively, showed partial or complete overlapping with previously reported κ-casein antihypertensive fragments (DQTEIPT, DKTEIPTINTIA, KTEIPTINTIA, TEIPTIN, EIPTINTIA, EIPTINTIAS, PTIN, and PTINTIASSGEP) [58]. Such peptides were purified from a κ-casein hydrolysate obtained using *L. helveticus* CEP cell envelope proteinases. Moreover, the other κ-casein-derived peptide, VIESPPEINTVQVTSTA, was previously reported as antihypertensive, together with other sequences sharing the epitopes VIESPPEIN and TVQVTSTA [46,58,61].

To the best of our knowledge, the two peptides deriving from serum albumin (DAFLGSFLYEYSR and RHPYFYAPELLYYANK), were not previously reported as anti-ACE peptides; nevertheless, serum album hydrolysates produced with different enzymes, such as papain and proteinase-K, were already reported as sources of different antihypertensive sequences [62,63].

In addition, the cell adhesion molecule 1 (GlyCAM-1), known as proteose-peptone 3 (PP3) [64], corresponding to the C-terminal-truncated variant of lactophorin [65] was already reported as source of angiotensin-converting enzyme (ACE)-inhibitory peptides. Three different GlyCAM-1 fragments were identified in the active subfractions of the fermented R-UF, and in particular, a large similarity was found between DETHLEAQPTDASAQ and a previously identified bioactive sequence isolated in RCEW hydrolysates obtained with commercial proteases [52].

Aiming at RCEW valorization through the reuse as food ingredient, the fermented R-UF fraction, enriched in bioactive peptides, was spray-dried and used as functional supplement for making fortified ricotta cheese, at a pilot plant level. The fortified ricotta cheese was characterized by a marked increase of the anti-ACE activity compared to the control and to the ricotta cheese samples obtained with the unfermented R-UF fraction at the same levels of fortification.

Based on the concentration found in the purified active fractions, it can be estimated that bioactive peptides corresponded to circa 14% (*w*/*w*) of the total peptide concentration of the spray-dried fermented R-UF. Consequently, circa 30 mg of bioactive peptides can be found in a 100 g portion of the ricotta cheese produced at a 5% fortification level.

Although the most recent literature shows a positive antihypertensive in vivo effect when the ACE-inhibitory peptides are used at doses of 3.8–52.5 mg/kg [66,67], an analytical quantification of the bioactive peptides in fortified ricotta cheese is needed to make further considerations. Moreover, in vivo and human studies are necessary to elucidate the dose-effect and the mechanisms involved in the activity of these sequences.

Compared to the use of unfermented spray-dried R-UF fraction, the fermentation reflected in a moderate acidification of the final product, especially at the highest level of inclusion in the ricotta cheese formulation (5% *w*/*w*). Obviously, the moisture of the ricotta cheeses was also affected by the addition of the dried RCEW fraction at the highest level of fortification. The supplementation also caused a relevant increase of the protein (calculated on the basis of the total nitrogen) and lactose contents, the latter was still abundant in both unfermented and fermented R-UF, although at a lower level. Probably due to the extensive proteolysis, the use of the fermented spray-dried R-UF attenuated the increase in hardness and chewiness that characterized the ricotta cheese fortified with the 5% unfermented R-UF.

Since peptides generally have a taste, covering the entire range of established taste modalities: sweet, bitter, umami, sour and salty [68], the sensory properties of the fortified ricotta cheese needed to be investigated. The fortification modified the overall characteristics of the ricotta cheese by decreasing the milk odor and taste, while increasing the acidic one. Moreover, the persistence of flavor and sapidity was also improved with the fortification.

## 5. Conclusions

A biotechnological protocol to obtain bioactive peptides with angiotensin-I-converting enzyme (ACE)-inhibitory activity was set up. A *Lactobacillus helveticus* selected strain was used as starter for the fermentation of the ultrafiltration protein-rich retentate (R-UF) obtained from the RCEW. The fermented R-UF was characterized by a high anti-ACE activity and the peptides responsible for the bioactivity showed partial or complete overlapping with previously reported κ-casein antihypertensive fragments.

When the freeze-dried fermented R-UF was used to enrich ricotta cheese at different fortification levels (1 and 5% *w*/*w*), a high anti-ACE activity was found. Indeed, a 100 g portion of the ricotta cheese produced at 5% fortification level containing circa 30 mg of bioactive peptides.

The fortification led to a moderate acidification, increased hardness and chewiness, and decreased the milk odor and taste of the ricotta cheese as compared to the control, while flavor persistence and sapidity improved.

The biotechnological protocol proposed here for the enrichment in antihypertensive bioactive peptides represent an easily scalable process that can be employed for the upcycle of the RCEW and its components, providing novel supplements for the design of innovative functional foods, with ricotta cheese as one of the many potential applications.

## Figures and Tables

**Table 1:** Characteristics of the RCEW inoculated (7.0 log10 cfu/mL) with selected LAB.

	RCEW					
	CT	FC13	B15Z	CR12	PR4	DIBCA2
pH	4.87 ± 0.05 ^a^	4.10 ± 0.04 ^c^	3.92 ± 0.04 ^d^	4.02 ± 0.05 ^d^	3.76 ± 0.05 ^e^	4.26 ± 0.05 ^b^
LAB density (log10 cfu/mL)	3.9 ± 0.1 ^d^	8.5 ± 0.2 ^a^	7.9 ± 0.2 ^b^	8.1 ± 0.1 ^b^	9.0 ± 0.2 ^a^	7.5 ± 0.1 ^c^
Peptides (mg/L)	364 ± 4 ^e^	703 ± 10 ^c^	775 ± 15 ^b^	819 ± 9 ^a^	795 ± 12 ^b^	525 ± 6 ^d^
ACE-inhibitory activity (%)	7.2 ± 0.2 ^d^	16.9 ± 0.2 ^c^	26.5 ± 0.2 ^b^	7.9 ± 0.4 ^d^	68.4 ± 0.6 ^a^	7.5 ± 0.3 ^d^

The data are the means of three independent experiments ± standard deviations (*n* = 3). ^a–e^ Values in the same row with different superscript letters differ significantly (*p* < 0.05). Fermentation was carried out at 30 °C (*L. delbrueckii* subsp. *bulgaricus* B15Z and *L. lactis* DIBCA2) or 37 °C (*L. casei* FC13, *L. helveticus* PR4, and *S. thermophilus* CR12). An uninoculated but incubated (30 °C) RCEW sample was used as control (CT).

**Table 2:** Proximal composition of ricotta cheese exhausted whey (RCEW), ultrafiltration protein-rich retentate (R-UF), and ultrafiltration permeate (P-UF).

	RCEW	R-UF	P-UF
pH	5.2 ± 0.1 ^a^	5.2 ± 0.2 ^a^	5.2 ± 0.1 ^a^
Total Nitrogen (mg/L)	60.02 ± 0.54 ^b^	290.02 ± 4.05 ^a^	1.34 ± 0.02 ^c^
Proteins * (% *w*/*v*)	0.38 ± 0.06 ^b^	1.85 ± 0.05 ^a^	0.08 ± 0.01 ^c^
Total Free Amino Acids (mg/L)	417 ± 5 ^b^	52 ± 2 ^c^	516 ± 9 ^a^
Lactose (% *w*/*v*)	3.80 ± 0.10 ^b^	5.19 ± 0.22 ^a^	3.42 ± 0.11 ^c^
Glucose (% *w*/*v*)	<0.01	<0.01	<0.01
Galactose (% *w*/*v*)	<0.01	<0.01	<0.01
Fat (% *w*/*v*)	0.16 ± 0.10 ^b^	0.75 ± 0.04 ^a^	<0.01
Ash *(%* *w*/*v*)	1.08 ± 0.12 ^b^	0.23 ± 0.08 ^c^	1.35 ± 0.11 ^a^

The data are the means of three independent experiments ± standard deviations (*n* = 3). ^a–c^ Values in the same row with different superscript letters differ significantly (*p* < 0.05). * Protein content is calculated as total nitrogen × 6.38.

**Table 3:** Characteristics of the RCEW fractions R-UF (prior fermentation) and fR-UF (after fermentation with *Lactobacillus helveticus* PR4 at 37 °C for 24 h).

	R-UF	fR-UF
pH	5.2 ± 0.1 ^a^	3.82 ± 0.2 ^b^
LAB density (log10 cfu/mL)	7.21 ± 0.2 ^b^	8.82 ± 0.4 ^a^
Peptides (mg/L)	65 ± 5 ^b^	3230 ± 25 ^a^
TFAA (mg/L)	50 ± 2 ^b^	896 ± 12 ^a^
Lactose (%)	5.2 ± 0.2 ^a^	3.5 ± 0.1 ^b^
ACE-inhibitory activity (%)	nd	88.2 ± 1.1

LAB, thermophilic presumptive lactic acid bacteria; TFAA, total free amino acids. The data are the means of three independent experiments ± standard deviations (*n* = 3). ^a,b^ Values in the same row, with different superscript letters, differ significantly (*p* < 0.05).

**Table 4:** List of the peptides identified in the partially purified peptide fractions obtained from the fR-UF through RP-FPLC. Fermentation was carried out with *Lactobacillus helveticus* PR4 at 37 °C for 24 h.

Fraction	Sequence	Mass (Da)	Length (aa)	Net Charge *	Hydrophobic Ratio (%)	NCBI Accession n° (Protein)
A	TIASGEPTSTPTTEA	1462.532	15	−2	38	CAF03625.1 (kappa-casein)
	DAFLGSFLYEYSR	1567.721	13	−1	44	P02769.4 (serum albumin)
	RHPYFYAPELLYYANK	2045.327	16	1.24	42	
	DETHLEAQPTDASAQ	1612.63	15	−3.75	32	ABY26541.1 (glycosylation-dependant cell adhesion molecule 1)
B	ILNKPEDETHLEAQPT	1835.001	16	−2.75	36	AAB27385.1 (PP3 homolog)
C	VIESPPEINTVQVTSTA	1884.118	17	−2	49	CAF03625.1 (kappa casein)
D	NQDKTEIPTINTIASGEPTSTPTIE	2657.869	25	−3	36	ACF15188.1 (kappa casein)
	AQPTDASAQFIR	1304.428	12	0	49	AAB27385.1 (PP3 homolog)

* The net charge was calculated at pH 7.

**Table 5:** Biochemical, nutritional, and textural characteristics of the ricotta cheeses.

	Ricotta cheeses				
	RC	RC1	RC5	RCf1	RCf5
Biochemical and nutritional characteristics					
pH	5.02 ± 0.03 ^a^	5.02 ± 0.02 ^a^	5.02 ± 0.03 ^a^	4.95 ± 0.03 ^b^	4.88 ± 0.04 ^b^
TTA (mL NaOH 0.1 M)	8.1 ± 0.2 ^b^	8.1 ± 0.2 ^b^	8.2 ± 0.3 ^b^	8.5 ± 0.1 ^b^	8.9 ± 0.3 ^a^
Moisture (%)	76.6 ± 0.5 ^a^	76.5 ± 0.7 ^a^	73.1 ± 0.5 ^b^	76.2 ± 0.8 ^a^	73.0 ± 0.5 ^b^
Proteins ^1^ (% *w*/*v*)	9.8 ± 0.5 ^b^	10.3 ± 0.6 ^ab^	10.9 ± 0.7 ^a^	10.3 ± 0.4 ^ab^	11.0 ± 0.6 ^a^
Fat (% *w*/*v*)	10.2 ± 0.3 ^a^	10.3 ± 0.4 ^a^	10.7 ± 0.4 ^a^	10.3 ± 0.3 ^a^	10.7 ± 0.5 ^a^
Carbohydrates (% *w*/*v*)	3.5 ± 0.3 ^b^	3.9 ± 0.4 ^b^	6.3 ± 0.2 ^a^	3.7 ± 0.3 ^b^	5.8 ± 0.3 ^a^
Ash (% *w*/*v*)	2.4 ± 0.2 ^a^	2.5 ± 0.3 ^a^	2.6 ± 0.2 ^a^	2.5 ± 0.1 ^a^	2.6 ± 0.3 ^a^
ACE-inhibitory activity	7.2 ±0.2 ^c^	7.5 ± 0.3 ^c^	7.5 ± 0.2 ^c^	25.1 ± 0.2 ^b^	64.3 ± 0.2 ^a^
Textural Profile Analysis					
Hardness (g)	244.9 ± 11.0 ^b^	244.1 ± 23.5 ^b^	357.3 ± 32.0 ^a^	242.5 ± 14.5 ^b^	253.2 ± 19.3 ^b^
Cohesiveness	0.519 ± 0.015 ^b^	0.468 ± 0.016 ^c^	0.566 ± 0.034 ^a^	0.584 ± 0.027 ^a^	0.601 ± 0.028 ^a^
Springiness	0.716 ± 0.016 ^a^	0.694 ± 0.011 ^b^	0.608 ± 0.036 ^c^	0.709 ± 0.010 ^ab^	0.627 ± 0.021 ^c^
Chewiness (g)	91.0 ± 3.5 ^a^	79.9 ± 5.2 ^bc^	100.4 ± 6.9 ^a^	63.6 ± 10.4 ^c^	85.0 ± 11.8 ^ab^

^1^ Protein concentration is calculated as total nitrogen × 6.38. The data are the means of three independent experiments ± standard deviations (*n* = 3). ^a–c^ Values in the same row, with different superscript letters, differ significantly (*p* < 0.05). RC, control ricotta cheese (without fortification); RC1 and RC5, ricotta cheeses fortified with 1 and 5% (*w*/*w*) spray-dried unfermented R-UF, respectively; RCf1 and RCf5, ricotta cheeses fortified with 1 and 5% (*w*/*w*) fR-UF, respectively.

**Table 6:** Sensory analysis of ricotta cheeses.

Attributes	RC	RC1	RC5	RCf1	RCf5
Visual aspect					
Color intensity	5.1 ± 0.2 ^b^	5.3 ± 0.4 ^b^	5.3 ± 0.3 ^b^	5.3 ± 0.4 ^b^	6.0 ± 0.4 ^a^
Color homogeneity	4.5 ± 0.2 ^a^	4.8 ± 0.3 ^a^	4.0 ± 0.3 ^b^	4.8 ± 0.3 ^a^	4.8 ± 0.3 ^a^
Odor					
Milk	5.0 ± 0.3 ^a^	4.8 ± 0.4 ^ab^	4.8 ± 0.5 ^ab^	4.5 ± 0.2 ^ab^	3.8 ± 0.4 ^c^
Acidic	1.1 ± 0.4 ^ab^	1.1 ± 0.4 ^ab^	1.1 ± 0.2 ^b^	1.5 ± 0.3 ^ab^	1.8 ± 0.4 ^a^
Flavor					
Sapidity	3.5 ± 0.3 ^b^	3.5 ± 0.4 ^b^	3.5 ± 0.4 ^b^	4.0 ± 0.3 ^ab^	4.3 ± 0.4 ^a^
Bitterness	0.5 ± 0.3 ^b^	0.5 ± 0.2 ^b^	0.5 ± 0.2 ^b^	0.5 ± 0.2 ^b^	1.1 ± 0.4 ^a^
Acidity	4.0 ± 0.3 ^c^	4.3 ± 0.3 ^c^	4.3 ± 0.3 ^c^	5.3 ± 0.4 ^b^	6.5 ± 0.4 ^a^
Sweetness	2.8 ± 0.5 ^a^	2.1 ± 0.2 ^b^	2.0 ± 0.4 ^b^	2.0 ± 0.3 ^b^	2.0 ± 0.3 ^b^
Milk	3.0 ± 0.4 ^a^	2.8 ± 0.4 ^a^	2.8 ± 0.5 ^a^	3.0 ± 0.2 ^a^	2.7 ± 0.3 ^a^
Butter	3.3 ± 0.6 ^a^	2.7 ± 0.4 ^a^	2.7 ± 0.4 ^a^	2.7 ± 0.4 ^a^	2.7 ± 0.5 ^a^
Off-flavors	0.7 ± 0.3 ^a^	0.7 ± 0.3 ^a^	0.8 ± 0.4 ^a^	0.8 ± 0.4 ^a^	0.8 ± 0.2 ^a^
Persitency	3.2 ± 0.5 ^b^	3.0 ± 0.3 ^b^	3.2 ± 0.4 ^b^	3.8 ± 0.5 ^b^	6.0 ± 0.7 ^a^
Texture					
Adhesiveness	2.7 ± 0.5 ^ab^	3.0 ± 0.2 ^a^	3.0 ± 0.4 ^a^	3.3 ± 0.3 ^a^	2.3 ± 0.0 ^b^
Graininess	2.5 ± 0.3 ^b^	2.8 ± 0.5 ^ab^	3.2 ± 0.4 ^a^	2.5 ± 0.4 ^b^	2.7 ± 0.4 ^ab^
Friability	3.2 ± 0.4 ^a^	3.3 ± 0.4 ^a^	3.3 ± 0.2 ^a^	3.3 ± 0.5 ^a^	3.3 ± 0.4 ^a^
Wetness	5.0 ± 0.2 ^a^	5.0 ± 0.4 ^ab^	4.7 ± 0.2 ^ab^	4.7 ± 0.3 ^ab^	4.5 ± 0.2 ^b^

The data are the means of three independent experiments ± standard deviations (*n* = 3). ^a–c^ Values in the same row, with different superscript letters, differ significantly (*p* < 0.05). RC, control ricotta cheese (without fortification); RC1 and RC5, ricotta cheeses fortified with 1 and 5% (*w*/*w*) spray-dried unfermented R-UF, respectively; RCf1 and RCf5, ricotta cheeses fortified with 1 and 5% (*w*/*w*) fR-UF, respectively.

## Supplementary Materials

The following are available online at www.mdpi.com/article/10.3390/foods10112573/s1, Table S1: List of microorganisms used in this study. The source of isolation and corresponding references are also reported, Table S2: List and definition of the attributes used for the sensory analysis carried out on ricotta cheese samples.
