# Supplementary material for: Antihypertensive Peptides from Ultrafiltration and Fermentation of the Ricotta Cheese Exhausted Whey: Design and Characterization of a Functional Ricotta Cheese
Source: Foods. 2021 Oct 25;10(11):2573. doi: 10.3390/foods10112573 (PMC8623162; doi:10.3390/foods10112573)
Supplement: Supplementary file 1 [file foods-10-02573-s001.zip › foods-1428598-supplementary.pdf]

Table S1. List of microorganisms used in this study. The source of isolation and corresponding references are also reported.

Strain	Medium	Temperature (°C)	Source of isolation	Reference
Lacticaseibacillus casei FC13	MRS	37	"Flor di capra" (sheep milk cheese)	[19]
Lactobacillus helveticus PR4			Parmigiano Reggiano cheese	[57] [21]
Lactobacillus delbrueckii subsp. *bulgaricus* B15Z		30	Caciocavallo Pugliese cheese	[20]
Lactococcus lactis DIBCA-2			Pecorino Umbro (sheep milk cheese)	[19]
Streptococcus thermophilus CR12	M17	37	Fermented milk	[22]

Table S2. List and definition of the attributes used for the sensory analysis carried out on ricotta cheese samples.

Attributes	Definition	Physical reference
Color intensity	Color of the ricotta samples	
Color homogeneity	Uniformity of the ricotta texture at visual inspection	
Odour		
Milk	Typical milk odor	Fresh milk
Acidic	Acid, sour, vinegar	Milk acidified with lactic and acetic acids
Flavor		
Sapidity	Sapidity taste	Ripened cheese
Bitterness	Bitter taste	Coffee
Acidity	Acid, sour, vinegar	Milk acidified with lactic and acetic acids
Sweetness	Sweet taste	Ricotta cheese added with sugar
Milk	Typical milk flavour	Fresh milk
Butter	Typical butter flavour	Butter
Off-flavors	Presence of off-flavours	
Persitency	After-taste persistency of the flavor	
Intensity	After-taste intensity of the flavor	
Texture		
Adesivnhess	Adherence to the spoon	
Graininess	Perception of grains (lumps) and heterogeneity in the mouth	
Friability	Tendency to break into smaller pieces	
Wetness	Capability of being melted in the mouth	
